# The molecular mechanism of SARS-CoV-2 evading host antiviral innate immunity

**DOI:** 10.1186/s12985-022-01783-5

**Published:** 2022-03-19

**Authors:** Wenjing Gu, Hui Gan, Yu Ma, Lina Xu, Zhangkai J. Cheng, Bizhou Li, Xinxing Zhang, Wujun Jiang, Jinlv Sun, Baoqing Sun, Chuangli Hao

**Affiliations:** 1 Department of Respiration, Children’s Hospital of Soochow University, Suzhou, 215003 China; 2 National Center for Respiratory Medicine, The First Affiliated Hospital of Guangzhou Medical University, National Clinical Research Center for Respiratory Disease, State Key Laboratory of Respiratory Disease, Guangzhou Institute of Respiratory Health, Guangzhou, 510120 China; 3 Department of Allergy, Peking Union Hospital, Peking Union Medical College, Beijing, China

**Keywords:** SARS-CoV-2, COVID-19, Antiviral innate immunity, Molecular mechanism, PRR

## Abstract

The newly identified Severe Acute Respiratory Syndrome Coronavirus 2 (SARS-CoV-2) has resulted in a global health emergency (COVID-19) because of its rapid spread and high mortality. Since the virus epidemic, many pathogenic mechanisms have been revealed, and virus-related vaccines have been successfully developed and applied in clinical practice. However, the pandemic is still developing, and new mutations are still emerging. Virus pathogenicity is closely related to the immune status of the host. As innate immunity is the body’s first defense against viruses, understanding the inhibitory effect of SARS-CoV-2 on innate immunity is of great significance for determining the target of antiviral intervention. This review summarizes the molecular mechanism by which SARS-CoV-2 escapes the host immune system, including suppressing innate immune production and blocking adaptive immune priming. Here, on the one hand, we devoted ourselves to summarizing the combined action of innate immune cells, cytokines, and chemokines to fine-tune the outcome of SARS-CoV-2 infection and the related immunopathogenesis. On the other hand, we focused on the effects of the SARS-CoV-2 on innate immunity, including enhancing viral adhesion, increasing the rate of virus invasion, inhibiting the transcription and translation of immune-related mRNA, increasing cellular mRNA degradation, and inhibiting protein transmembrane transport. This review on the underlying mechanism should provide theoretical support for developing future molecular targeted drugs against SARS-CoV-2. Nevertheless, SARS-CoV-2 is a completely new virus, and people’s understanding of it is in the process of rapid growth, and various new studies are also being carried out. Although we strive to make our review as inclusive as possible, there may still be incompleteness.

## Introduction

A novel coronavirus disease, COVID-19, has created a global pandemic in 2020, posing an enormous challenge to healthcare systems and affected communities. COVID-19 is caused by severe acute respiratory syndrome-coronavirus-2 (SARS-CoV-2) that manifests as bronchitis, pneumonia, or a severe respiratory illness. Virus pathogenicity is closely related to the immune status of the host. The body’s immunity, including innate immunity and acquired specific immunity, is the key to resisting virus invasion. Innate immunity is the body’s first defense against viruses, and it is also the basis for the body’s specific immunity [1].

The immune response during SARS-CoV-2 infection goes through two main phases: a protective phase based on immune defense, while the second is characterized by extensive inflammation. Some SARS-CoV-2-infected patients become severely ill because the virus suppresses the host’s immune response in the first stage and produces an inflammatory storm in the second. So, the basic approach in treatment management is to boost immunity in the first stage and suppress it in the second stage. This review mainly focuses on how SARS-CoV-2 inhibits the host’s innate immunity and delays the onset of adaptive immunity during the first stage after it infects the host.

### Viral adhesion

Like any virus, the SARS-CoV-2 life cycle is divided into three stages; entry (attachment, fusion, uncoating), genome replication (replication and protein translation), and exit (assembly, maturation, and release) [2]. The first step for a virus to invade a host is viral adhesion. Viral infection relies on cell entry, using host mechanisms to replicate copies of the virus, which the host then releases. The entry of viruses into host cells is mainly mediated by S protein, which makes viruses attach to host cell receptors, promotes the fusion between host and virus membrane, and enables the successful entry of viruses into host cells [3, 4]. Several receptors are involved in the adhesion and invasion of SARS-CoV-2 in the human body, and Angiotensin-converting enzyme 2 (ACE2) is a recognized receptor. Other possible interaction sites such as arginine–glycine–aspartic (RGD) motif and DPP4 are also being studied extensively. In addition to ACE2, SARS-CoV-2 infection requires dozens of host factors, such as arginine–glycine–aspartic (RGD) motif and DPP4, which are also being studied extensively. SARS-CoV-2 uses clathrin-mediated endocytosis to gain access into cells is also a key aspect of virus infectivity. Whole-genome CRISPR screenings have identified that endocytic trafficking regulators, such as SNX27 and retromer, are required for SARS-CoV-2 infection[5].

### ACE2

ACE2 is currently recognized as the SARS-CoV-2 receptor, which plays an important role in virus adhesion and invasion. The three-dimensional crystal structure of the SARS-CoV-2 spike receptor-binding domain (RBD) complexed with its receptor, human ACE2, has also been solved [6]. ACE2 is expressed throughout the body’s vasculature, allowing SARS-CoV-2 access to multiple organ systems [7, 8]. There are two forms of ACE2, Full-length membrane ACE2 (mACE2) and a soluble form sACE2 [9]. The mACE2 is the receptor site for the spike (S) protein of SARS-CoV-2 and may cause an increase in AngII and further activation of the AngII/AT1R axis, worsening inflammation. As for the soluble form, studies showed that an elevated level of sACE2 correlates with disease severity, possibly due to an increase in AngII [10, 11]. There are also studies showing that the elevated sACE2 level may be evidence of the increased expression of mACE2 and can also increase the activity of ADAM17, thus increasing the susceptibility of SARS-CoV-2 [12]. However, some researchers have suggested that elevated sACE2 levels may have a protective effect because they inhibit SARS-CoV-2 from binding to mACE [9]. The S protein on the SARS-CoV-2 envelope is composed of S1 and S2 subunits, and the S1 protein/receptor interaction is a key determinant of host species infection by SARS-CoV-2 [13].

Since ACE2 is widely expressed in human organ tissues, including nasal and oral mucosa, vascular system, gastrointestinal tract (GI), this allows SARS-CoV-2 to infect the human body in various ways [14, 15]. The nasal epithelium is the main route of SARS-CoV-2 infection. Studies have shown that the expression of ACE2 in the nasal epithelium of older children (10–17 years old), adolescents (18–24 years old), and adults (> 25 years old) is higher than that of young children (4–9 years old), which may be one of the reasons why young children are not susceptible to this virus or have relatively mild symptoms after infection [16]. Although the expression of ACE2 in the lungs is very low, most patients with SARS-CoV-2 infection present with respiratory distress. It is considered that SARS-CoV-2 causes a series of inflammatory responses, such as the release of interferons, which enhances the expression of ACE2 and thus enhances the infection [17, 18].

### RGD

The ubiquitous arginine–glycine–aspartic (RGD) motif is well known for its role in cell attachment and cell adhesion. The RGD motif is the minimal peptide sequence required for binding proteins of the integrin family, which are commonly utilized as receptors by many human viruses [19]. Viral proteins with RGD motifs promote infection by binding integrin heterodimers, activating phosphatidylinositol-3 kinase (PI-3 K) or mitogen-activate protein kinase (MAPK), thereby facilitating virus-cell attachment and infection [20]. Studies have shown that RGD may play a role in the adhesion and invasion of SARS-CoV-2 [21, 22]. The RGD motif may bind integrins in a parallel or sequential ACE2-independent manner, playing an important role in the rapid spread of SARS-CoV-2 [23]. Ca^2+^ ions will enhance the binding of the viral spike protein to the host cell, which will promote the virus’s entry into the cell for infection, replication, and pathogenesis [22].

### Dipeptidyl peptidase 4 (DPP4)

DPP4, also known as cluster of differentiation 26 (CD26), is the receptor of Middle East Respiratory Syndrome Coronavirus (MERS-CoV) and has been recently proposed as a potential drug target for COVID-19 [24]. Vankadari et al. showed that human DPP4/CD26 might interact with the S1 domain of the SARS-Cov-2 spike glycoprotein [25]. They described a complex model of docking between SARS-CoV-2 spike glycoprotein and DPP4, showing the large interface between these proteins, which is very similar to other coronaviruses that use DPP4 as a functional receptor, such as CoVHKU4 and MERS-CoV, suggesting that in addition to ACE2, other molecules on the cell surface can influence viral adhesion.

### Endocytosis

Endocytosis was a common way for the virus to enter the host cell and this process was a key aspect of virus infectivity. In the SARS-CoV-2 infection process, it used its S protein to interact with the cell surface, after engagement with the plasma membrane, SARS-CoV-2 undergoes rapid endocytosis [26]. Bayati et al. found that by deleting clathrin heavy chain in cells, clathrin-mediated endocytosis was blocked and viral infectivity was reduced [26]. Those data demonstrated SARS-CoV-2 spike protein is internalized by clathrin-mediated endocytosis. Besides ACE2 mentioned above, many other molecules are involved in the endocytosis mechanism of SARS-COV-2. NRP1 was also reported as a SARS-CoV-2 receptor and played an important role in CendR-associated endocytosis mechanisms [27]. Previous data had shown ACE2 receptors were the main way for SARS-CoV-2 to target the lung and enter the body. Non-muscle myosin heavy chain IIA (MYH9) was identified as an ACE2 coreceptor, it could colocalize with SARS-CoV-2 S-protein at the membrane and enhance SARS-CoV-2 entry through endocytosis [28]. Jia found that SNX27 mediated the endocytosis pathway of SARS-COV-2. SNX27 can mediate the endocytosis of S-protein, promote its surface expression, and enhance the transfection effect of S pseudovirus [5]. Interaction of Fc regions of antibodies on the surface of the virus with Fc receptors of target cells is the other way of endocytosis. While Fc receptors express were stimulated by viral particle endocytosis into the cell [29]. Above all, the fate of SARS-CoV-2 in the cell is determined both by the type of receptor and intracellular transport pathway.

### Suppression of innate immunity

After viral infection, the mammalian innate immune system acts rapidly to detect and block viral infection at all stages of the viral life cycle. Human innate immunity plays a key role in maintaining health by protecting it from coronavirus. Its level is regarded as an alternative solution before introducing effective drugs and vaccines [30]. Shi et al. proposed two phases during the immune responses induced by SARS-CoV-2 infection [31]. The first immune defense-based protective phase of SARS-CoV-2 infection, closely associated with innate immune responses, is very important. Moving from the evidence that innate immune cells and pro-inflammatory cytokines were the main actors in innate immune response, critical for the eventual outcome of COVID-19 infection. The innate immune cells could recognize the virus and trigger a local innate response to viral clearance.

Dendritic cells, macrophages, neutrophils, and NK cells are the first defense line to initiate an immune response and affect its type and intensity. The frequency of monocytes, macrophages, and neutrophils was higher in the BALF of SARS-CoV-2 infection groups. Compared to mild cases, the percentage of macrophages and neutrophils was higher, and mDC, pDC, T, and NK cells were lower in severe patients [32]. The inhibition of SARS to innate immune cells is mainly shown in the following aspects.

### NKG2A and apoptosis of NK cells

Studies have confirmed that innate lymphoid cells (ILCs) were lower in adults COVID-19 inpatients, especially in severe cases, and ILC abundance was inversely proportional to the probability and duration of hospitalization and the severity of inflammation [33, 34]. Cao et al. demonstrated that the total number of NK cells decreased significantly in the severe COVID-19 cases compared to non-severe ones [35]. The CD94/NK group 2 member A (NKG2A) is a heterodimeric inhibitory receptor expressed by NK cells [36]. It can bind to non-classical HLA class I molecules (HLA-E) and inhibit NK cell toxicity and cytokine secretion through two inhibitory immune receptor tyrosine inhibitor molecules [37]. Studies have shown high expression of NKG2A in COVID-19 patients. Upregulation of NKG2A was associated with the exhaustion of NK cells at the early stage of SARS-CoV-2 infection, and therefore, was associated with severe disease progression [38]. Bortolotti co-cultured spike protein transfected lung epithelial cells with NK cells and found that SARS-CoV-2 spike-1 protein (SP1) expression in lung epithelial cells led to reduced degranulation of NK cells.

Meanwhile, when SP1 is expressed in lung epithelial cells, NK cell inhibition receptor NKG2A/CD94 regulation is increased. It is suggested that SP1 is expressed in lung epithelial cells after SARS-CoV-2 infection and affects NK cells through HLA-E/NKG2A interaction. NK cell depletion may be involved in the immune pathogenesis of SARS-CoV-2 infection [39]. NKG2A has been widely studied in cancer. Blocking NKG2A in cancer can restore NK function, thus regulating tumor growth. However, SARS-CoV-2 up-regulates the level of NKG2A on NK cells, leading to the failure of the immune response against viral pathogens, thus overruling the host’s innate immune response [40]. Therefore, the use of NKG2A inhibitors is expected to restore the body’s innate immune function in COVID-19, thus achieving therapeutic effects.

### ORF8 and immune evasion

SARS-CoV-2 open reading frame 8 (ORF8) was one of the most rapidly evolving beta coronavirus proteins. It had a hypervariable gene that could facilitate the virus adaptation to the human host [41]. The structure, function, and sequence variation of ORF8 may be pivotal for SARS-CoV-2 as a deadly human pathogen. Thomas G and his colleagues founded SARS-CoV-2 ORF8 had a special crystal structure, through covalent disulfide-linked dimer and separate noncovalent interface form unique large-scale assemblies, which can make ORF8 as a rapidly evolving accessory protein mediating unique immune suppression and evasion activities [42]. SARS-COV-2 ORF8 can mediate immune evasion through down-regulation of MHC-Ι [43]. The study showed that ORF8 protein had a function of impairs the antigen presentation system and in ORF8-expressing cells, MHC-Ι molecules are selectively targeted for lysosomal degradation. This results in cytotoxic T cells not being able to completely clear SARS-COV-2 infected cells. In Singapore, researchers found a SARS-COV-2 variant with 382 nucleotide fragments missing in the ORF8 gene fragment (∆382), and that patients with the variant had generally a mild disease and required less oxygen therapy than those infected with wild-type virus, suggesting that ORF8 played an important role in the pathogenicity of SARS-COV-2 [44]. ORF8 as a secreted protein, which antibodies could treat as the principal markers of SARS-CoV-2 infections [45]. Inhibited of antiviral signaling pathway was an important way to get rid of host innate immune. ORF8 was screened as the major viral proteins that showed strong inhibition on type I interferon (IFN-β) and NF-κB-responsive promoter [45]. ORF8 also through the IFN-I signaling impacted intracellular immunity and through the mitogen-activated protein kinases (MAPKs) impacted growth pathways [46].

### Reduced number and impaired function of DCs

When infected with SARS-CoV-2, DCs were significantly reduced with function impaired, and ratios of conventional DCs (cDC) to plasmacytoid DCs (pDC) were increased among acute severe patients, which could explain the reduction of the interferon production and the early decrease of innate immunity against SARS-CoV-2 infection [47]. In addition, compared with healthy donors, CD80, CD86, CCR7, and HLA-DR were significantly induced in pDCs, cDC1, and cDC2, implicating that the maturation of DCs was influenced by SARS-CoV-2 infection [47]. Zhou et al. demonstrated that the functionality of COVID-19 patient-derived DCs would induce proliferation of allogeneic CD4 and CD8 T cells through in vitro tests. Thus, even if abundant neutralizing antibodies against the RBD and NP were detected in SARS-CoV-2 infection patients, T cell response was remarkably delayed because of the reduced number and impaired function of DCs [47]. Compared to healthy cases, the expression of IFN-α, mainly produced by pDC, was undermined in SARS-CoV-2 infected patients [48]. Another study analyzed the peripheral blood samples from SARS-CoV-2 infected patients and found that the cDCs were significantly depleted in those diagnosed with acute respiratory distress syndrome (ARDS) [49]. A previous study found that antigen-presenting cells (APCs) infected with MERS-CoV lead to a lack of DC signals, resulting in the inefficiency of T cell response to the virus and promoting NK cells attended in apoptosis [50]. MERS-CoV has been demonstrated to infect Mo-DCs, rapidly inducing high expression levels of IFN-γ, IP-10, IL-12, and RANTES. Recently, studies showed that SARS-CoV-2 infection downregulated the ACE2 expression in moDCs [51]. Investigations also found that MoDCs were permissive to SARS-CoV-2 infection and protein expression but did not support productive virus replication. They demonstrated that SARS-CoV-2 attenuated immune response in moDCs through antagonized STAT1 Phosphorylation. Subsequently, Parker and colleagues described the repertoire of human leukocyte antigen class II-bound peptides presented by HLA-DR diverse monocyte-derived dendritic cells pulsed with SARS-CoV-2 spike protein, hoping to understand and elicit protective immune responses to SARS-CoV-2 [52]. Based on those studies, DCs were regarded as a critical role in revealing how the host defends itself against invasion by SARS-CoV-2. Recently, Han et al. proposed that the dendritic cell-based therapeutic approach may be a potential strategy for SARS-CoV-2 infection [53].

### Excessive inflammation of macrophages and neutrophils

Macrophages and neutrophils might act as pro-inflammatory immune cells, contributing to excessive inflammation resulting in systemic manifestations and multiorgan dysfunctions, especially in severe COVID-19 patients with severe symptoms. Toor et al. also identified that hyperactivation of macrophages and neutrophils led to ARDS even subsequent death in COVID-19 cases and through tempered macrophage plasticity helped control and manage SARS-CoV-2 infection [54]. Macrophages produce pro-inflammatory cytokines after detecting a broad range of pathogen-associated molecular patterns (PAMPs) using pattern recognition receptors, such as toll-like receptors (TLRs). Shirato K had reported that the SARS-CoV-2 spike protein S1 subunit strongly induces IL-6 and IL-1β production in murine and human macrophages by activating TLR4 signaling through c-Jun N-terminal kinase (JNK) and nuclear translocation of nuclear factor-κB (NF-κB) pathway y[55]. P44/42 MAPK and Akt pathways release pro-inflammatory factors in macrophages [56]. Infection of SARS-CoV-2 through CD147 initiated the JAK-STAT pathway, further inducing expression of cyclophilin A (CyPA); CyPA reciprocally bound to CD147 and triggered MAPK pathway.

Consequently, the MAPK pathway regulated the expression of cytokines and chemokines, which promoted the development of cytokine storms [57]. Thorne et al. discovered that activating cytoplasmic RNA sensors RIG-I and MDA5 triggers a robust innate immune response when SARS-CoV-2 invades. The inflammatory mediators produced during epithelial cell infection can stimulate primary human macrophages to enhance cytokine production and drive cellular activation [58]. Neutrophils of COVID-19 patients acted as a hyper-inflammation driver using increased cytokine secretion and cell degranulation [59]. It was also observed that HLA-DR was impaired on the surface of SARS-CoV-2 infected neutrophils and PD-L1 expression increased, resulting in a decrease in Ag-presentation capacity. However, reduction of Ag presentation was also observed on monocytes, and the alteration in monocyte phenotype was presented at different disease stages in COVID-19 patients [60, 61].

### Inhibits interferon production

In the innate immune system, host cells rapidly open multiple signal cascades by recognizing pathogen-related molecular patterns (PAMPs), leading to the transcriptional induction of type I and type III interferons (IFNs) [62, 63]. Bastard et al. and Zhang et al. had demonstrated that IFN signaling was important in defense against SARS-CoV-2. They also observed that limiting IFN signaling pathway leads to severe COVID-19 [64, 65]. Chen et al. reported that low production of IFN-γ was associated with severe cases of COVID-19 [66]. In addition, in the established SARS-Cov-2 infection mice model, type I, type II, and type III IFN did not seem to be up-regulated. While type I IFN signaling is required to recruit pro-inflammatory cells into the lungs and ISG expression, not for viral clearance. So, these results indicate that type I interferon cannot inhibit SARS-CoV-2 virus replication but can drive important pathological reactions [67]. Recently, it has been reported that MDA5 signaling pathways which induce the IFN response govern the innate immune response to SARS-CoV-2 in lung epithelial cells [68].

Further exploring the regulatory mechanisms of IFN-pathway signaling underlying SARS-CoV-2s infection, the JAK-1/2-induced STAT that transduces signals downstream of the IFN-α receptor, including STAT1, were significantly elevated. Interestingly, the Janus kinase inhibitor (JAKi) Ruxolitinib could neutralize SARS-CoV2 mediated complement activation, indicating that the IFN pathway is associated with the severity of COVID-19 patients [70]. Collectively, the hampered IFN pathway is the main mechanism of the high pathogenicity of SARS-CoV-2.

Previously researchers had observed that the cytokine storm existed in SARS-CoV or MERS-CoV severe cases [71]. Recently, Wang et al. confirmed that the cytokine storm also appeared in severe patients infected by SARS-CoV-2 [72]. Immune cells like NK cells, macrophages, DC, neutrophils, monocytes, and tissue-resident cells as epithelial and endothelial cells contribute to the SARS-CoV-2 cytokine storm. High pro-inflammatory cytokines and chemokines, such as IL-1β, IL-2, IL-6, IL-7, IL-10, TNF-α, IFN-γ, G-CSF, CCL2, CXCL10 were detected on COVID-19 patients. Thus, the over secretion of pro-inflammatory cytokines by SARS-CoV-2 infection leads to severe outcomes.

These findings indicated that the immune cells provide critical insights into the innate immune recognition and signaling response to SARS-CoV-2. All in all, these findings provide critical insights into the innate immune recognition and the molecular basis of signaling response to SARS-CoV-2. Therefore, innate immunity is essential for the early control of infection. In addition, we should further understand the molecular mechanism of some viral proteins inhibiting immunity.

SARS-CoV-2 encodes a wide range of viral structural proteins and non-structural proteins (NSP) with diverse functional roles in viral replication and packaging that affect the IFN signaling pathway and, in turn, impair the IFN-mediated antiviral responses [4]. The most important proteins are NSP1, NSP8, NSP9, and NSP16, which play an important role in inhibiting host transcription, translation, and protein transport.

### Non-structural proteins1 (NSP1)

Nsp1 is encoded at the very 5′ end of ORF1a and is the first coronaviral protein produced in infected cells. Prior studies have shown that Nsp1 plays an essential role in suppressing host translation on SARS-CoV-1 and inducing mRNA cleavage and decay [73], leading to cell-intrinsic innate immune responses inhibited such as interferon signaling interrupted [74–77]. Jiang et al. [78] screened all the viral proteins of SARS-CoV-2 for the protein–protein interactions by a mammalian two-hybrid system. They discovered that Nsp1 of SARS-CoV-2 has the most interacting partners among all the viral proteins and likely functions as a hub for the viral proteins. The mechanism that SARS-CoV-2 Nsp1 inhibits translation has remained poorly understood until recently. Schubert et al. [65] and Thoms et al. [68] provided insights into how Nsp1 binds to the 40S subunit of the ribosome and blocks the mRNA entry channel. Using cryo-electron microscopy, the two studies highlight areas of interaction between Nsp1 and the ribosome and show the 5′UTR of the viral transcript. SARS-CoV-2 Nsp1 causes translation inhibition by sterically occluding the entrance region of the mRNA channel in the free 40S subunits, the 43S pre-initiation complex, and in empty, non-translating 80S ribosomes [79]. Mendez et al. [80] found that central regions of nsp1 do not participate in docking into the 40S mRNA entry channel nonetheless stabilize its association with the ribosome and mRNA, enhancing its restriction of host gene expression and enabling mRNA containing the SARS-CoV-2 leader sequence to escape translational repression. The latest literature shows that the translation output of reporters containing full-length viral 5′untranslated regions (UTRs) is significantly enhanced, which could explain how Nsp1 inhibits global translation while still translating sufficient amounts of viral mRNAs [79]. However, it would seem that the effect of Nsp1 is restricted to translational inhibition with little if any, direct effect on immune response gene transcription and mRNA stability [81].

### Non-structural protein 8 (NSP 8) and non-structural protein 9 (NSP 9)

During infection of human cells, SARS-CoV-2 NSP 8 and NSP 9 were found to be essential for replication by binding the Signal Recognition Particle (SRP) and disrupting protein trafficking [82, 83]. The SRP is a universally conserved complex that binds to the 80S ribosome and acts to co-translationally scan the nascent peptide to identify hydrophobic signal peptides present in integral membrane proteins and proteins secreted from the plasma membrane [84].NSP8 and NSP9 were observed that could bind to the 7SL RNA in the signal recognition particle and interfere with protein trafficking to the cell membrane upon infection [85]. Consistent with this, the study shows that NSP8 and NSP9 localize broadly throughout the cytoplasm when expressed in human cells or upon SARS-CoV-2 infection [83, 86]. Disruption of SRP leads to a global reduction in puromycin levels in the cell membrane and suppresses protein integration into the cell membrane in SARS-CoV-2 infected cells.

Furthermore, by antagonizing membrane trafficking, SARS-CoV-2 may prevent viral antigens from being presented on MHC and allow infected cells to escape T-cell recognition and clearance. In this way, interference with these essential cellular processes might further aid SARS-CoV-2 in evading the host immune response. At the same time, the study reveals that NSP8/9-mediated viral suppression of SRP would act to suppress the IFN response upon infection [83].

### Non‐structural proteins16 (NSP 16)

Nsp16, a 2′-O-methyltransferase (2′-O-MTase), forms part of the replication-transcription complex and plays an essential role in mRNA translation, virus replication, and escape the host cell innate response system [87–89]. Nsp16 achieves this by mimicking its human homolog, CMTr1, which methylates mRNA to enhance translation efficiency and distinguish self from other to escape IFIT-mediated suppression [90]. NSP16 binds to the 5′ splice site recognition sequence of U1 and the branch point recognition site of U2. Consistent with U1/U2, Banerjee et al. observed that NSP16 localizes within the nucleus upon SARS-CoV-2 infection in human cells and disrupts global mRNA splicing in SARS-CoV-2-infected human cells, and inhibition of mRNA splicing suppresses host interferon response to viral infection [83]. Several studies have shown that Nsp16 facilitates the transfer of a methyl group from its S-adenosylmethionine (SAM) cofactor to the 2′ hydroxyl of ribose sugar of viral mRNA [91]. This methylation improves translation efficiency and camouflages the mRNA so that it is not recognized by intracellular pathogen recognition receptors, such as IFIT and RIG-I [90, 92]. Due to the important role in SARS-CoV-2 replication, NSP16 is one of the highly viable targets for drug discovery [93].

**Fig. 1:**
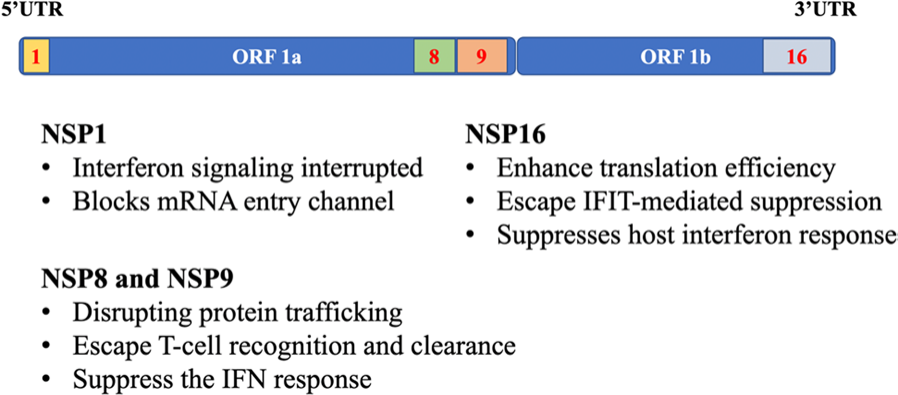
Non-structural proteins (NSPs) and their functions

Except for NSP 1, NSP 8, NSP 9, and NSP 16 (Fig. 1), other kinds of NSP play an important role in immunity and inflammation of the body. Recently, Xu et al. [94] found that NSP12 could promote the activation of RIPK1 and lead to systemic inflammatory response, known as cytokine storm, which may lead to death. NSP12 323L variant that carries a Pro323Leu amino acid substitution in NSP12 showed increased ability to activate RIPK1, suggesting that inhibition of NSP12 may provide a therapeutic option for the treatment of COVID-19. Russo et al. revealed that the NSP3 macrodomain reverses PARP9/DTX3L-dependent ADP-ribosylation induced by interferon signaling to counteract the host innate immune response [95].

### Inhibiting adaptive immune priming

In COVID-19 patients, there was some evidence of an inverse correlation between T cell abundance and disease severity. DiPiazza et al. observed higher CD8 +/CD4 + T cell ratios in mild cases, whereas the opposite trend was found in severe cases [96]. SARS-CoV-2 infection may resist the body’s antiviral immunity by destroying the function of cytotoxic lymphocytes in the early stage [38]. The count of CD8 + T cells was reported to be decreased during COVID-19 infection, and, in severe cases, memory CD4 + T cell and T regulatory cell count were significantly reduced [97]. Lymphocyte counts, CD3 + T cells, and CD8 + T cells were significantly higher in patients with mild stages and/or only mild symptoms than in patients with severe disease [35].

SARS-CoV-2 restrains antigen presentation by downregulating MHC class I and II molecules and, therefore, inhibits the T-cell-mediated immune responses [98]. It has been revealed that cytotoxic CD8 + T cells exhibit functional exhaustion patterns, such as the expression of NKG2A, PD-1, and TIM-3. In addition, CD8 + T cell degranulation was reduced in COVID-19 patients compared with healthy donors, resulting in reduced production of IL-2, IFN-γ, and granzyme B [38]. All these indicate that SARS-CoV-2 has a strong ability to suppress the adaptive immune response.

In addition to cellular immunity, SARS-CoV-2 also inhibited humoral immunity. It was reported that the response of effector B cells to viral infection was related to the severity of the disease. Matthew et al. observed the B cell antigen-receptor sequencing data and demonstrated that SARS-CoV-2-specific responsiveness was broad, with the participation of IgM, IgA, and IgG antibodies. However, the serum antibody responses against the RBD of the SARS-CoV-2 spike protein were higher in outpatient than ICU patients [99]. Other studies have shown low levels of antibodies in people who have died from COVID-19. Anti-S and anti-RBD IgG levels peaked quickly in discharged patients and showed good virus clearance, while those who died took longer to produce high levels of antibodies [100], which may be related to the number of effector memory (EM) Th cells. Golovkin A confirmed that the levels of Th cells in the peripheral blood of COVID-19 patients were reduced compared to healthy people, while there was no difference in neither absolute number nor percentage of Th cells in patients with moderate and severe disease. However, the number of EM Th cells was significantly lower in severe patients than in moderate patients [101]. To further subdivision the relative numbers of IFNγ-producing Th17-like and Tfh-like cell subsets were decreased in patients with moderate and severe infections. The impaired Tfh-like cell differentiation in patients with COVID-19 could lead to the maturation of dysfunctional B cells and the alteration of the humoral immune response during acute COVID-19. These all lead to a significant reduction in B cells in the COVID-19 patient, and the reduction in B cells is related to the severity of the disease.

### Authors’ opinion

**Fig. 2:**
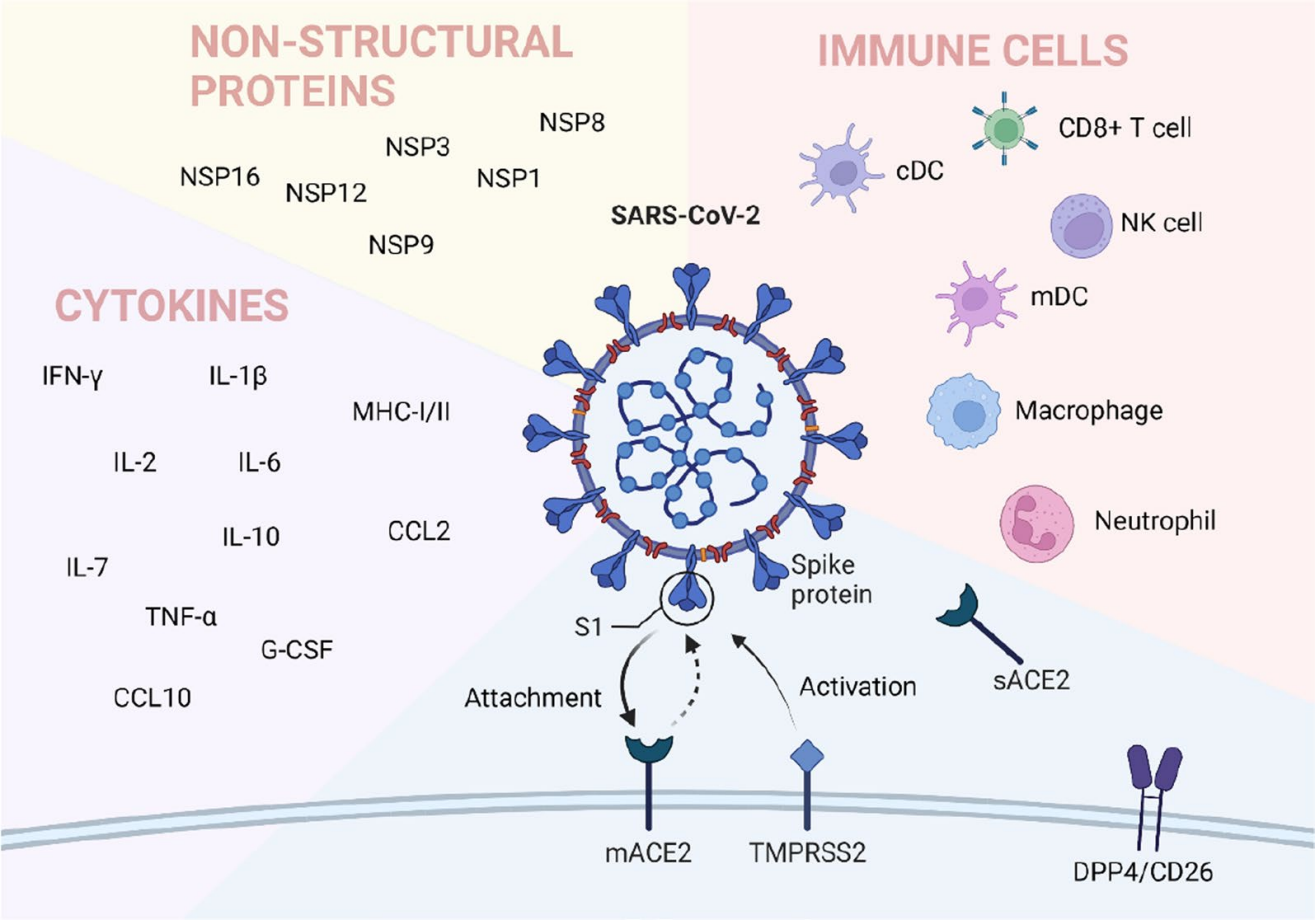
SARS-CoV-2 bypasses human innate immunity through multiple mechanisms. Created with BioRender.com

SARS-CoV-2 bypasses multiple innate immune activation pathways through distinct mechanisms, as shown in Fig. 2. Innate immunity, as the first line of defense for humans against viral infections, is very fragile under the attack of the new coronavirus, allowing the virus to spread quickly, cause disease, and even lead to death. Despite the launch of vaccines and new drugs, the COVID-19 pandemic is still developing. We still need more efficient and practical prevention and treatment methods. Blocking the virus from breaking through natural immunity may be a new starting point. Therefore, we reviewed the suppression of innate immunity by the SARS-CoV-2 to provide ideas for developing new preventive and therapeutic drugs. As shown in Table 1, SARS-CoV-2 invades the body, replicates, and releases viral particles in various ways. For each way of invading and destroying the body, it is possible to develop specific antibody drugs or small molecule inhibitors. In fact, with the passage of time, more and more drugs have been registered and served in the clinic setting [102–105]. From this perspective, it is only a matter of time before humans control the new crown epidemic. Moreover, this time may not be as long as imagined.

**Table 1:** The potential route of SARS-CoV-2 to evade host innate immunity and the potential target for future drug development

Mechanism	Potential route	Potential target [69]
Viral adhesion and invasion	ACE2 (mACE2 and sACE2)	Entry inhibition: Arbidol Chloroquine/hydroxychloroquine Camostat Convalescent Plasma/immunoglobulins
	Arginine-glycine-aspartic motif	
	Dipeptidyl peptidase 4	
	Endocytosis	
	ORF8	
Suppression of innate immunity	Dendritic cells	Inhibiting consequences of cytokine storm: Tocilizumab Sarilumab Ruxolitinib Baricitinib
	Macrophages	
	Neutrophils	
	NK cells	
Affect the IFN signaling pathway	NSP 1	Inhibition of translation and protease inhibitors: Lopinavir/ritonavir Niclosamide Darunavir Interferon beta
	NSP 8	
	NSP 9	
	NSP 16	
	NSP 12	
	NSP 3	
Inhibiting adaptive immune response	Low CD8 +/CD4 + T cell	Inhibition of viral polymerase: Ribavirin Favipiravir Remdesivir
	MHC class I and II molecules downregulated	

## Data Availability

Not applicable.
